# Supplementary material for: Rational design of T-DNA vectors enables predictable, single-copy integration in Arabidopsis thaliana
Source: bioRxiv. 2026 Jul 5:2026.06.08.730999. Originally published 2026 Jun 10. Preprint. [Version 2] doi: 10.64898/2026.06.08.730999 (PMC13277942; doi:10.64898/2026.06.08.730999)
Supplement: Supplement 2 [file media-2.pdf]

Extended Data Table 1: Transformation counts

Figure	Description	Line	Seed weight (mg)	Seed count	RUBY-	RUBY+	Total	Tx RUBY- (%)	Tx RUBY+ (%)	Tx total (%)	Fraction RUBY+ (%)	RUBY+ per 10000 seed
Fig. 1i	Starter vector	AWS429	900	45000	730	58	788	1.62	0.13	1.75	7.36	12.89
Fig. 2b	Wild type	AWS493	400	20000	257	28	285	1.29	0.14	1.43	9.82	14.00
Fig. 2b	Border Var 1	AWS494	400	20000	419	40	459	2.10	0.20	2.30	8.71	20.00
Fig. 2b	Border Var 2	AWS495	400	20000	394	37	431	1.97	0.19	2.16	8.58	18.50
Fig. 2b	Border Var 3	AWS496	400	20000	326	26	352	1.63	0.13	1.76	7.39	13.00
Fig. 2b	Border Var 4	AWS497	1000	50000	12	90	102	0.02	0.18	0.20	88.24	18.00
Fig. 2b	Border Var 5	AWS590	400	20000	302	35	337	1.51	0.18	1.69	10.39	17.50
Fig. 2b	Border Var 6	AWS499	600	30000	30	91	121	0.10	0.30	0.40	75.21	30.33
Fig. 2e	RB-Max	AWS619	400	20000	226	9	235	1.13	0.05	1.18	3.83	4.50
Fig. 2e	RB-Mid	AWS620	400	20000	32	69	101	0.16	0.35	0.51	68.32	34.50
Fig. 2e	RB-Min	AWS621	400	20000	8	32	40	0.04	0.16	0.20	80.00	16.00
Fig. 2g	OD 2.0, p-RB-Max	AWS601	400	20000	115	4	119	0.58	0.02	0.60	3.36	2.00
Fig. 2g	OD 2.0, p-RB-Mid	AWS602	400	20000	40	72	112	0.20	0.36	0.56	64.29	36.00
Fig. 2g	OD 2.0, p-RB-Min	AWS603	400	20000	12	47	59	0.06	0.24	0.30	79.66	23.50
Fig. 2g	OD 0.8, p-RB-Max	AWS607	400	20000	215	8	223	1.08	0.04	1.12	3.59	4.00
Fig. 2g	OD 0.8, p-RB-Mid	AWS610	400	20000	29	50	79	0.15	0.25	0.40	63.29	25.00
Fig. 2g	OD 0.8, p-RB-Min	AWS613	400	20000	6	22	28	0.03	0.11	0.14	78.57	11.00
Fig. 2g	OD 0.2, p-RB-Max	AWS604	400	20000	165	6	171	0.83	0.03	0.86	3.51	3.00
Fig. 2g	OD 0.2, p-RB-Mid	AWS605	400	20000	30	68	98	0.15	0.34	0.49	69.39	34.00
Fig. 2g	OD 0.2, p-RB-Min	AWS606	400	20000	14	29	43	0.07	0.15	0.22	67.44	14.50
Fig. 2h	EHA105, p-RB-Max	AWS439	400	20000	72	6	78	0.36	0.03	0.39	7.69	3.00
Fig. 2h	EHA105, p-RB-Mid	AWS441	400	20000	19	5	24	0.10	0.03	0.12	20.83	2.50
Fig. 2h	EHA105, p-RB-Min	AWS442	400	20000	0	1	1	0.00	0.01	0.01	100.00	0.50
Fig. 2h	LBA4404, p-RB-Max	AWS625	400	20000	30	23	53	0.15	0.12	0.27	43.40	11.50
Fig. 2h	LBA4404, p-RB-Mid	AWS626	400	20000	1	2	3	0.01	0.01	0.02	66.67	1.00
Fig. 2h	LBA4404, p-RB-Min	AWS627	400	20000	0	0	0	0.00	0.00	0.00	0.00	0.00
Fig. 2h	AGL-1, p-RB-Max	AWS628	400	20000	69	7	76	0.35	0.04	0.38	9.21	3.50
Fig. 2h	AGL-1, p-RB-Mid	AWS629	400	20000	39	17	56	0.20	0.09	0.28	30.36	8.50
Fig. 2h	AGL-1, p-RB-Min	AWS630	400	20000	1	4	5	0.01	0.02	0.03	80.00	2.00
Fig. 2i	WS, p-RB-Max	AWS608	400	20000	91	15	106	0.46	0.08	0.53	14.15	7.50
Fig. 2i	WS, p-RB-Mid	AWS611	400	20000	20	34	54	0.10	0.17	0.27	62.96	17.00
Fig. 2i	WS, p-RB-Min	AWS614	400	20000	0	10	10	0.00	0.05	0.05	100.00	5.00
Fig. 2i	Ler, p-RB-Max	AWS609	400	20000	25	4	29	0.13	0.02	0.15	13.79	2.00
Fig. 2i	Ler, p-RB-Mid	AWS612	400	20000	10	24	34	0.05	0.12	0.17	70.59	12.00
Fig. 2i	Ler, p-RB-Min	AWS615	400	20000	2	0	2	0.01	0.00	0.01	0.00	0.00
Fig. 2i	C24, p-RB-Max	AWS529	400	20000	28	4	32	0.14	0.02	0.16	12.50	2.00
Fig. 2i	C24, p-RB-Mid	AWS531	400	20000	5	8	13	0.03	0.04	0.07	61.54	4.00
Fig. 2i	C24, p-RB-Min	AWS532	400	20000	2	7	9	0.01	0.04	0.05	77.78	3.50
Fig. 3e	pRB-Mid (WT)	AWS340	400	20000	44	64	108	0.22	0.32	0.54	59.26	32.00
Fig. 3e	pRB-Mid (R106H)	AWS339	400	20000	86	69	155	0.43	0.35	0.78	44.52	34.50
Fig. 3e	pCAMBIA (WT)	AWS338	200	10000	81	9	90	0.81	0.09	0.90	10.00	9.00
Fig. 3e	pCAMBIA (R106H)	AWS337	200	10000	138	9	147	1.38	0.09	1.47	6.12	9.00
Fig. 5a	pRB-Mid (1xLB)	AWS313	400	20000	65	94	159	0.33	0.47	0.80	59.12	47.00
Fig. 5a	pRB-Mid (2xLB)	AWS317	400	20000	54	76	130	0.27	0.38	0.65	58.46	38.00
Extended Data Fig. 7d	pUBQ10, Divergent	AWS429	200	10000	231	16	247	2.31	0.16	2.47	6.48	16.00
Extended Data Fig. 7d	pUBQ10, Reverse	AWS430	200	10000	202	28	230	2.02	0.28	2.30	12.17	28.00
Extended Data Fig. 7d	pUBQ10, Forward	AWS433	200	10000	142	24	166	1.42	0.24	1.66	14.46	24.00
Extended Data Fig. 7d	pUBQ10, Covergent	AWS434	200	10000	79	31	110	0.79	0.31	1.10	28.18	31.00
Extended Data Fig. 7e	RUBY	AWS500	300	15000	183	17	200	1.22	0.11	1.33	8.50	11.33
Extended Data Fig. 7e	Linker	AWS501	300	15000	264	20	284	1.76	0.13	1.89	7.04	13.33
Extended Data Fig. 7e	Recoded	AWS504	300	15000	222	17	239	1.48	0.11	1.59	7.11	11.33
Extended Data Fig. 7e	Intron	AWS553	200	10000	107	16	123	1.07	0.16	1.23	13.01	16.00
Extended Data Fig. 7f	pRPS5A, Divergent	AWS427	200	10000	197	13	210	1.97	0.13	2.10	6.19	13.00
Extended Data Fig. 7f	pRPS5A, Reverse	AWS428	200	10000	175	32	207	1.75	0.32	2.07	15.46	32.00
Extended Data Fig. 7f	pRPS5A, Forward	AWS431	200	10000	157	24	181	1.57	0.24	1.81	13.26	24.00
Extended Data Fig. 7f	pRPS5A, Covergent	AWS432	200	10000	151	44	195	1.51	0.44	1.95	22.56	44.00
Extended Data Fig. 7g	tHSP	AWS643	200	10000	160	10	170	1.60	0.10	1.70	5.88	10.00
Extended Data Fig. 7g	tFAD	AWS644	200	10000	114	6	120	1.14	0.06	1.20	5.00	6.00
Extended Data Fig. 7g	tNOS	AWS645	200	10000	134	12	146	1.34	0.12	1.46	8.22	12.00
Extended Data Fig. 7g	tOCS	AWS646	200	10000	120	26	146	1.20	0.26	1.46	17.81	26.00

Extended Data Table 2: iPCR sequencing results

Line	Border	Alignment	Sequence
2	LB	Chr3:21578067:21578910	CGTCGCAGGCACGTCTTACAGTGGTTTACACCACAATATTTCAAATTCAACTTGTAGCATTTGCCATCGACTTAAAACTT<br>TTAAATAAAATATTTTAAAATTAATATATGCAAGTTCTACAAGTTGATTTTGACCACTACATGTTGGTCTACAGAGAAATG<br>AAACTATGAATTTTAATATCCTTTCTTGGAATGAAAGGTTCCTTTTCGTACTCAAATTTATATTATAAAATTTTGATTCAAC<br>AGTTTCATCATAATCACTTACTCTCTGCTTTAATTTTAATTTCGTATTCAAAACCGCAGGTGGATGAAGACAAATTAGCAG<br>AGACCAAAACGTAGTTGAGGATGAAACAATTACTTAGCAGAAAATGAACAGAAACAGAGGAGTCTTACTTATTCACCA<br>AGAAACTCTTATATCACTTAATTTTCTCAAGTTAAAAATAGGATATGTATATGTTTTTTAACTCTACCTAAAAGGAAGATA<br>CTGAAATGAGGAAAGCTGAAATAAAAAGACTAGAAGTAGGAAACACTATTCCTCATTAAACAATGGAGAAGACTCAAA<br>TCAAGAAATCACAACTTCATCTTAAAATCTCAGTCAAACAACATCAATGAAGCCGAAGCTGCAGCTTTCTCTTTCTCCAG<br>GTCGGCTTCAAGTTCTGTCACTGCCAGCTTTCGCTTCTTGACCTGTTTCTCGAGTTCTCCGATCCGTGTGTCGTAAGCTA<br>TCTGTTTTTCCAAAGCCTGATCAAACTTCTGACGCAACCAGTCCAAGTTCAAGCCTGCTTTAATCAGATCCGTAATCGTA<br>TTGTCTGCTTCGTTCATGTCTTCCACTGTGAGTTCCTTAGTAGATTTGGACAGTGTCTTCGTAAGGTCAAGAAGAGCAT<br>GCATAGCATCTTGTTACCGACCAATGCTCAGAACTCACGACTCC
2	RB	Chr3:21571044:21578058	AACTTCTCTCAAATACACACCGCGTGCCGTTTAGTGCTCTGCTTATTGGACAACGTGTGGTATAACAGAACGTGAACGC<br>TTAAACATTTTTCAACTGGGCCGGGCTCTTTGGCCCAACTCTATGACTTGAATGATACCATCAGTGAAGTCAGGTCGGT<br>CGGTGTTCGTCGTATCAAAGAAGTTTCCTTTAGAGCTTTTTCTCTCATTCTGTCTTCGTCTAAAAAGGTGAAATCTTTAAC<br>AACTGATCATATTACTCTTAATGATTTCGTAATCTCCTGTTCGTTACGTTGATTATTGTTATCTGTTAAAATTGATGTTTAA<br>GAGTGTCATCGTACTACTGGTAGCTTGATTGCAGATAACAATGGGGAAAGAAGTTGATAACAAGTTCACTTGGGTGAT<br>TAAGAATTTCTCCTCTCAGCAGTCCAGGAAAAATTATTCTGATGAATTCTTCGTCGATGGCTGCAAATGGTAAGAAGTG<br>AATTTTTTCGAATGATTTCATCAATTTAGTTTTTTTTTTGTTTTCCGTGAAATTGAGGATTGACTAAATTTTCTTGAAGGCG<br>TCTTCTGGCCTTTCCTAAAGGAAATGGTGTTGAGAAGTTGTCACTGTATCTGGCCGTTGCTGGTAGTGAATTTTTACCT<br>GATGGATGGAGAAGACACGCATATTTCCACTTCAGTGTAGTGAATCAACTCTCTGATGAACTCTCTCAAGCTAGAGGT<br>GATTGTTTTTCTACACAATGCTCGTTACTTGATATGCGTTGGAAATTGATTGCAACAGTTTTTTTTTTCCTTATGTTGTTTG<br>AGTTGGTTGGTTTAAGACTAGTGTCAATGTTTCGTAGCAAGTTTAGAGCATGGTTCATGGATGGCTTTATTTTATGTGAA<br>GTGAAGTCAATGATGAAATGGAATTTATTTCGTTGTTGTTACAGAAACAAAAAATTGGTTTGATGCGAGTACTAGTGAC<br>TGGGGTTTTACATCAATGCTTTCCCTTAAAAAACTTCATGACAAAGATGGTGGATTCCTGGTTAATGGAGAGCTCAAGA<br>TTGTTGTCGATGTTTCTGTTCTTGAAGTTATTGGCAAATTAGACGTACCAGTGGAATCTGAAGAGACAACCACCAAAGC<br>TCTAAGCGAGCTAGAGGAAAATGACGTACCAGAGGAATCTGAAGAGACAACTAAAGCTCTAAGCAAGGTAGACGAA<br>AACGATGGTGCAGAGTCTAATGATTCGCTCAAGGAAGCTTCATCAGTAAAGGAAAGCATGGATGTTAATGGCTTTCGA<br>GTTCTTCCTTCACAGGTATGAAATCAAGAATGGTGTAGTTGTTTCTTAAAATATCTAACCGACATATGTTCTATTCTGTTT<br>TGTGAATTGTTTCTCAAGTAGCAGAGAGCACACTTGTATATTCAGTAAAAACAAGAAATGAAATTGTAAAAGTTTCTTGA<br>TACATGTATGATGTATCTATGATTTGCAGAATATGAGCGAAACTGGAATATTGGAGATGTTTTTGTCTGTTCTGATTTATT<br>CAAGTCTCTGATAACTGCCTCCTCAATTTGTTTTTTTCTACTTAATTCATCTTTCTATCACTTTCAGGTGGAAACTGTGAGC<br>TGTATATTTGAAAGACACCCCGACATAGCATCAGAGTTCGGTCCAAAGAATCAGCATCTGAGGTCAGCTTACATGAAC<br>GTCCTTCTCAGCCTAATCAAGACTATGTGCCAGTCGACTCAGGAACTTTCCAAGGATGATCTGTCAGACGCAGATGCT<br>GCTCTTGCATACTTGACGGATGCGGGGTTGAATCTAAATTGGTTGGAGGAGAAACTGGAAGAAGTGTCAGAAAAGAA<br>GGAGAACGAGGAAGCTGGTGAGACTCGGGTGCATGAAATTGAAGAAGAGTTGAAGGAATTAAAGCTGAAGTGCTCA<br>AACCTGGAAGCTCAGCTGGAGAAGGAGAAAGCAGATGTGTCTGTGGCCAGAGCTCCTTTCTCCTTCGATGATATTGTT<br>TAATGGCTTCAGTATTCAGATTCCTTCTTATAGTTCTGTTTGGTGTTTTAGTCCTCTTGAAACTCTGCAAGATAAGTGGCT<br>TAGGTTGGATGTTTCCGCATTTTGTGGCTCTTGACTCGTTAGGTTTCTTTAATCCAGTGTGTTTCTTGTAAAGAAGTATTG<br>TGGTTCTAAGACTATTAGTCAGATTTTTTGGTTGTTTCGTTTAGGTGTGTTTCTTTTGAGTAAGGATCATTTTTTCCTTCTC<br>TTTTCTAACTAAACAGATGGGTAACGTTCACATATGGATCAAGTAACACAAAAAAAAATCACTTTATCATTGAATTTGACT<br>ATTTCTAAATATGTAAAATTACCTAACAATTACCATTACCAAATCTTTGGGGTTAAAGTATCAAAATATATACTGGGTTCT<br>GTTCATGGTAATTATACGTATGAAGAGGACTAGAGGAGTGTGACGGTACTGCAAACGAGGTCTACAACGGGCAGGG<br>ATTGAACCAAAATTGTAGGAAACCAAAAAACAAAAAATAGAGATAATTTGATTGTTAATCGATTAATTATTAGACCAGAA
5	LB	Chr2:15943893:15950062	TACTTTAGCATTCAGTTTCGTAAGACCTTGACCTCCTCTTTAACTTTGGTTATGTAGTATGGTTCTAACTTATATTCTTTGT<br>CTAGAATTGAAATTTACGCTCTCTTCTGTTCAGGGTTTCGGGTTCTTGGCAATATCAGCGTCACCAATACTTTCAGGCAA<br>TGGACAAAAGGGCCTGTTCTACGTAGCGCTAACGGTTATATCCGTTGGTATATTTGGACGGTCTATCTCTTTGGGAGTA<br>TTTACAGAAGACCAACTAGAAGACGGTAGGAACAAGGGAAACCCCGCCAAGCTCGTTAGCTTTGTCATTGGAAATGT<br>GGGGAATTTTGTGTTCTTGTTACTTGCTGCTATTGCAATGCCTCAGATAAGTCCGTGGTTTGTTAGGTTCACGATCCCCT<br>CGGGCTGTGAGGTATTAGCAATGCTGATTTTTATTTCCGGTGCGTGTTCATACAAACGGGTTAAACCAGGTGGGAGCC<br>CTTTGACAACGGTTTTCCGGGTCTTTATGGCTTCTGCTTCCAAAATGTCTTGTGCATACTCGAACAACTCAAGCCAGCTC<br>TATGAAAAAGCTGAGTGTGATCAAGATATTAAGCCCCACACAAGCAGCTTAAGGTTCAATAAAAATTCACTTCATTAAG<br>AGTTGTTAACCCACACTACTGTATAACACTCGTAACTCTTGATTTTTTCAAACACAGGTATCTAGATAGAGCTGCAATGA<br>TACTCCAAACAGAGTCTTTGGAACAACAGAGGAAAAACAGATGGAAGCTTTGTCGGGTGACAGAAGTGGAGCAAAC<br>GAAAAGCGTCATACGCACGGTTCCTTTGTTTGCGACATCTCTCATTTCGGGGATTGTCTTCTCTCTTGGTAATACTTTCTT<br>CCTTGAACAAGCAAACCATATGGACTCCAAGTTCGGATCATGGAACCTTCCTCTCCCACTCCTCTTACTCTTTAGTGAAG<br>CTGCAAGGCTAGGATCCCGAGAACTATGTGTCATGGCTGCAAAACGCCATGCCATTGACTTCCCTGAATCACCTAAAC<br>AAACCAAAACGCCTTATGGTATACCAGTCTCCATCATCCTATCGATATTTTGCTGCTCCATCGCTGCACATGTTGAGTCT<br>AGGAGGCTCAAAGTAGTTAGTACTCAAGGTTTACTACATGAAACTGTCCCCATGAGTGTATTCTGGCTGCTCCCACAGT<br>ACATTCTACTCGGTTCAATCACAGGAATCTACGAAAACAGCTTTGCGCTTTACCTAGAAGAAACGGTGCCTGAAGAGTT<br>GAGCCAATACATGGTTCTATTAAACGTGGGAGTGTGCGGGGTCGGGATCATGAGCAACATTGCTCTTGTTAGTTTGGT<br>GGGCAGTGTAAGCGGAGGCAAATGGTTTCAAGACACAATAAACAAGAGCCGGGTGGATAACTATTATTGGGTTATAA<br>CAGTGTTTTGCATGTTCAATCTCTTGTTATATTTCATTGTAACCTATAGGTACACAGTGTGTAACAAGAAAGATGGTGCA<br>ACACAAGAAAATGATCGAAGGATAATCGCTTCTGTGTAGTACTTGTGTTGTGTGCCCAATGTTTCAATTATACATGTATG<br>AGGTTAGATGAGGTTATTGCGTTTGTGGCTTCGGAAAAACATAGAAGAGAAATTAAAAAGGAATTCTCTCAATGTTTTT<br>GACCAAATCCTCAAATCATCTTCAGGCAACTAAACCATGTTTGCACACACATTAATAAACCAAAGACTAAATGGGACTG<br>GGACATCAGCTCACTTTAAAACTGAACCATTAAAACCTTCAATGACTTTTAGAAACAAATAAAGTAAGTTATGACTTAAA<br>TTGGAGGACAAGATTTAGAAAAACAAATCGAATTTTATGGAAAATAAAGAATTTAGTCCAAAATAGATTTACAAGAATA<br>ATTGGGAATCAACGGTCTTCCTTCAAATTCAAATTTGCAATTACATGGAGATACTTCTCGTATTACTCACAGTCACAAGG<br>CATCAAAATGGAACCCACCGAACATCAAGTTAAAGAACAAATTCACTAAAATTTTGAAAGTTTCCTAAATCTTAAAAAAA<br>GTAACTGCAAACTATTATTCTAGTTAAATAATCAGAAAGTCATTTTAAAGCCTGATACCTCTAAATACCATCATTATCTTT<br>GCCTATGTTCGAACCCCTTTGATGAGTGTTAAAATGTTGCTCTTATGAAAATCACATTATTATTTTAGTATCTATCCAAAG<br>GTTGGAATAAAAAGTTATTTTCTTCTGAATCTAATTGTACTGTGTATGGTTGGAAAGAAGATACAATACAAAAGATGAAC<br>AAAAAAAAAAAAAAAAAAAAAATGTTAATTTCCCAAGATGAGGATTTAAAATTGCCAACTTGGTTGAGGAACAAAAGCAT<br>ATCAATCATTGTGGCTTTTTATTTATGGTCTTTCTTTAGACTCTCTGTAGATTCTCATTCTCTTGTCAACTTCGAACTTCAG<br>ACAGTCCTATTAAATAAAACAGTTGGGAAAATAAAAGTAATTGAAAACTAAATTCTAATTACATAAGAAAAAGGGTTTA

5	RB	Chr2:15942019:15943852	CATCTCCCATTGGCTAACAAGATTTAAGGCGGTTATAAGGGTACTTCGGTAATTTGCTATTGGTGTTGACATTTAACCTT<br>TTGGGCACATGAGTTGTTTGTTTGTATATCGAGATACTTTCTCTTTTTTTCCTAAGGGGAAATGATTTTGTGACAATGAAA<br>CGTTTAATACTATATAAATTTGGATCATAATCTCGATAACAAAAAGCATTGGAGAATGTGGTTAAACTAGCATGCTAGG<br>AAACATAAAGGGGCATATCCTATCACAAACTATACTTATCTACGAAAATATGAATTACAAAATAAATAAATAAAATGAAC<br>TACAAAATTTAGTACAATGCCAAAATACGTATAAACTACAAACCCTAACAAAATTTTACAAAAATAGTTGTACTCATCTAA<br>ATCAATATTGATCTAAAAGGCGTTAATTTTTTGCATTGACATGTGACAGAATAAATTGCACAAGAAATGGCAATAAATAT<br>TTTTAGGCGTACTATTTGCTAAACCGACATTAAAGAACTGTCAAACAAATATAATTTTAATATGCAATGATGATTCCTTGC<br>TTAGCTTCTTGGTTCAACGGCCACAGCAAAAAATTCAACATCAAGGAAGTAATTAAATAGTATAGTAAATGTTTAGTGAT<br>CATAATTGTGTGTCTAAGATAACAAGGTCGTAATAATGGAGTTTAGCGAGAGAGAGGTCGAACCTTAATTATTAATTAA<br>CGTATGAAATATAATCATGATGATATTCTAACAACATTTTTTTTTTGTTGTGTCTGCACATTCTAACAATATCTAAATTCCA<br>AAAACTGGTACTAAGGTTTATTATGAGTTATGACCATAAGAATTTACGTAGCATATATATAAGATGATTTTCCGAAATTTC<br>AAGAATAATTTCTTTATAGGAAATCTTTAGAGTTAAATCAGTTTTTTGTTTTCTTTTTGTAACTCGACTAATTTAATTTATGT<br>ATAGTTTACTACTATAAGATTAATGTATATTCGAAACACATTATGAACGGCAATCACACAAAACGTAAATTAAATACATA
6	LB	Chr5:22771295:22776455	GATTCAAATCTCAACCTCTTCTTGTCTTCCAAGTCTGGCTTTAACACTGTTTGTATGCAAACAGTTGCACATGTCTTTGTT<br>TACTTTGTTTTAGATGCTTCTCTCAGTGTCATCAGCATTCAAGTGCGTTTTAAGTCAGTAACAAGATGAATACCTCTTGTT<br>GGCATCTTAAATCACACGAATTGTTCATGTTAAAGAAGAAGTTTCAAATTGTTTGTTGATATTGAAGAGAATGAGAACTA<br>TTTTCTATGTTTACTTTATTTTGCTATTTTCATTTTTTTCTTTCAGTTCCCTACTCTTATCTGTCTTGTGATTCCAAATGCAGA<br>ACACACACAGAATCTCCCATGGCTGCATTTGCAACGGCAGAAGCGTGTGACAGCAACGCAGAACTAATATCAAATGG<br>AGACTTACGCGCTCTTCACCCAATCTTCAAGATCTATGGCCAAAGAAGATGCTTCTCAGGACCAATCGTGACTCTCAAG<br>GTCTTTGAAGACAATGTCCTCGTCAGAAACCAACTAGAAACGAAAGGAGAAGGCGGAGTCTTAGTTATAGACGGTGG<br>TGGAAGCATGAGATGCGCGCTTGTTGGAGGAAACCTCGGACAGTTAGCTCAGAACAACGGGTGGTCGGGGATTGTT<br>GTGAATGGATGCGTTAGAGATGTGGATGAGATCAATGACTGCGATGTTGGGGTCAGGGCATTGGGATCTAACCCGTT<br>GAAATCTACTAAGAAAGGTCATGGTGAGAAGAATGTGCCGGTTCATATTGGAGGAACTTTGATTAGAGATGGAGAGT<br>GGCTATATGCTGATAGTGATGGTATCTTGATCTCCAAGACCGAACTCTCTGTTTGAGTCAGAACGTTTCCGTGATAGTG<br>CTAAGAGACTACAGCTTCTTGGAGCAAGGTGGTTTATTCGAATTCTTAAGAATCTGTTTAATACTCCAAATAATCATTTTT<br>ATTACAATATACTTACGTTTTATGAAATTACAAAATAAAAGTCTATTTAGTTCAACAAGTTTGGATCATGTGATATGTATA<br>AGAAACTTTCATTGAATTGAAAGAGATCAATAAGTCAAAACATGTGTATCAGTGAATACATTATACAACAAGGATATAAT<br>TTCTAAAGATGTTATTTTCGTCTTCCAGCATTGTAATGATTCTTGTACGAATGGATTTTAGTTATTTTGAAATTTAATTGAT<br>AGACATGTATAAATTATAATTGTATACACAAGAGCGTACTATTTTACTTGAATTTGAATACAAAATTCTGCTATGAGGTTT<br>GAAACTTGAAAGAATACATATCGTGGTGGACCAATTACTACTTTTATGGAATTGAAACAAATGAATGAATACTATTTCAA<br>AATTTGACCAAAAAAATAAAAACACCATTTCAAACTAAAACAAAATTCATACCGAACCAAACAAAACAAGATCAGATTTA<br>CTTGTCACGTTAATTTCGGTTCACTCATTTTTCTCGACCAACACACTTCGAAATCGTACGGAAAAATCGGTGCTCCCCGT<br>TAATAAAAAAAAATAAACGGTGTTTCCCAAGAATAATAAAAAGAATAAAAACATATATATTATTCAATGCTAGAGAAACT<br>ATATTATTAATATGTTCCGTCACATTAATAAATAAAAGCATCATAAAAAGTTAGACAGACCACACCATCACCATGTTCAT<br>GCCATTGATAATTTGACGTATATGTGGACATTTCATGTTTTCTCTTTAACACTCCTTTGTTTTTATACATAGGATCTACAAT<br>AAGTCTATTATTTCTAGCATATTTTGTATCTATCGAATCTTCTCAATCAAAAGTTAAATTATATGTTGAAATATGTCTTTGAT<br>CACATATTGATGTTGTAAAATAATTTGGATGATATGACACTCTAGACTAAAATAAGTAAATTCTAGACCATACAAATCTAT<br>AATTGATTCCAATTAGACACGAATATATTATACCACGACCACTTGTTTCTCAATCTAATTTGATGGAACTTATTAATCAAA<br>AGCTAAAAACGGCTGCTCTTTAGATTTATCATTGCGATTAAGAAGCTATAATTTGTTTTTCTTGTTTTGGTGTCTTGCTTG<br>AATCCATTACTTATCAATTTGTCATTCATTACTTCACCAAAAAAAAAAAAAAGAATAGTGTCGTGTAGCGAAGGAATTATG<br>GGACCTCCAATAAGTCCATAGGGCATGGAATATGACTTAACGAGGAAAATGATGTTTGACTAAAACCATAATTTCACGT<br>TTCCACAAAATGGCACTATCCTCTCTTATTTAATTCTATGATTTTTCTTAATTTCTCTTAATAAGGTGATGATTTAACGATAA<br>TTCTGGCTGTTGATACATTCGTTTTAAAAAGATCAAAATATGAGATATCTCATGGTTTATACTTTATAGAACTTGTAATTTT<br>GAGAAAAAGTGGTTATGAGTGCCTACATAATTCTATATGCACTAACATATACATGACCAACTTTTTCAAACAATTGGATC<br>CTTTTTTTCGTAAACATTTGGCTTGCTCTCAAAAACAGTCAAGACATCATGTTCTTATTTATTTTCACCTTTAAAAAGGACA
6	RB	Chr5:22776467:22778231	TGAAACGTATTAACCTAATTAAACTGCAATATAAGGAGATACGTTTACCAACCATTGTGAAATTCAGATATTCAAATATA<br>GATTGAATCTAATATATATAACACCTATTAAGTAGTTAAGTACTGGTTAAAGAAACCTAAAATAAAATCATTAAACGAAC<br>TAAATAACATCAGACAAATATTCTTTACAAATATATGAGATACGTTAACGCTTATATCTTTTCTTTTCTTTCTTACATGTTAA<br>CGTTTATAGTCTGAAAAGAGGAAGAAACAAAATGACTAACGTGCGTTAAGGATGATCTAATCTAATTGTATGTAGAGA<br>CATTTAATAAAATTGGGAATAATCAATCAATGACTTTTAATTATACCTTAAATTGAATATTAAATTGGAAATTGAGGAGGC<br>AGTTTTTCCAAAAAAAAACGGTCAAAAAAGAAACATTGACCAAGCCTCTTTTAGTATTTTGTTTTTCGCTTTCACTTTTATT<br>CTTCTTTGTCTTTTTACGAGCCGCAAAATATCCCGTTACGGAGCTGTGACCTTACACTCATGTGACAGCCAAAGCACGG<br>TTGTGGCGTGGTGTACCTTTTTTTTTTTTTTTTTTTTCTTCTTACAGATATTTTTATTTTACATCGTGGGGCCCTCAAAACGA<br>ACTTTGAAAGGACGGTGGATCGACGGCTCTGATGTCTTGTCAGTTTTAAGTATTCAATTATCCATGTAAACCGATCCCA<br>TCCGAACCAAAAATCGAACTGTTATCTAGGGGCTACTAGGCTCCCATTTCCACTGTGTAAACTTGGTTTAATTTCTACAT<br>CACTTGGCCCATTAGGCCCATTATAGCTTAATAAAAATAAAATATTTGGAATGGACCCAGCAGACTTGTTTTGACTGTCC<br>GATGATCGAACCAATAGTCAATAGAGAAACATGTCAACGATCCGACCCGAGAAAAGCGGGTCGGATTGAAAATACCC
14	LB	Chr5:1397972:1398927	GCTTCTGTTTAAATCTTTTACTACTTTCAGTTTTATATCTCTGATAAACTCTATAATATTGACTGATTCCTAAAGGACACTG<br>TCTCAGGATCTATCCCCTACATTCTTGTTCTACCTTGATGGATCATATATTTTACCTTAGTAAAGAAAATCGTCCAAGAAA<br>TTCATCATTAATTAAAAACATACATATAACATAGTCCTTCTTCGAATATAAGACTTCAATTCCTCAAAAAAAAATTAAACGA<br>AATAAATTATTTTTAAATATATATTCAAGAAACTGATGTTCATCGAGTTTAATTATTCAGTTGAATTACAATTCTTACTTTTA<br>GAAATTTGTATTGCGACCAAGAAGAAACATTCTTTAAGCTCAAAGCTTCATATAATTTACACATACGTAATTTATAGATAA<br>CACGTTGACCGAGAGTATTAGAAGCTCACAGTGCTTATAGGAAAGTCAAAACTGACTGCATCAGTACGATTTTTACGT<br>GTTCGTTTATGGATTATATAATACAAAATAGCTTATACATCAGTTTTAAAAAAATTAAACAAATTAATTAAGATGTAAAAC
14	RB	Chr5:13576233:13577280	TTTCTTCTCAATTAGGGTTCTCCCAATTTTCAATTTCATTTCCTTTGATATTAGGGTTCTTTCGAGTTAGTGTACGAATTAG<br>GGATCTTTACTAATTTCAGTTCTATGTATTTCAGATCTTGCTAAGAACTTATATAATTCAACGCTTTCTTTCAAAGGTTTGT<br>CCAAGTCGATAGATTTCTTATGCCTTTGATTTCTTACACAAAAACTTTATTGCGAATGTGATTGTTTTTTGTATCATACATC<br>TTGTGTGTTGCTGTCTGACTTGTTTTCTTTCTTATACATGTTTTGTGTTGCTGTCTGATTCATGCTTGTTTTGGGATACTGT<br>CTCAACGGTTCAAAATAAGGATAACAGATAAAATTAGGCCTCGACATAAAAACCGTACTCGCATACCCGAACCGGAAC<br>CGAACAAAAAAAAACGATTCAGGGCGGGTACGAGTATAAGGATTTTACACTGATGGGTCCTATGTAAGTGTACCCGT<br>GGGTATCGGTTCGGGTTCGGGTATTACCTGATATCCGAAAGGGTAAACCGAACACCCGAACCAAACACATTAACTCAT<br>TAACCACCCTTAACCAGTTAACCCTTATGTCGATTTAGGTTTTTTACTTTTGTTGATTTAGCTTCTTCCCTAACCCCTACTT

15	LB	Chr3:982891:986543	CCTATGTGAATACAACTTGTAATGGTTTTGCTTAAAATGTGAGAACATAACCATGTGAGGAAAATTTTGTTCATGTATGC<br>CCCACACTTTTAGACACCATTGTTTTCAATTGATAAGTCAAGTTTGAAGTTTCAACTCACTCACAGATCTATATATACAGA<br>AGAAACGAAGTCTTTTAACATCATCAACTTACAAGCAATCTAGAATCTCTCTCATTTGTATCCATACATCTTCACAAGCTT<br>CAAGAATCAAACTTCAAGACAAGAAAATGGCTTTGGTGAGAAGTCTCTTTAGCGCAAAGAAGATTCTTGGCGGTTCTTT<br>AGTAAAAACAAGCAAGGCACCGCCAAAAGGGTTTCTTGCAGTGTACGTCGGCGAGAGCCAGAAGAAGCAGAGACAT<br>TTTGTACCAGTCTCATACTTGAACCAGCCTTTGTTTCAAGATCTTCTAAGCAAATGTGAAGAAGAGTTTGGTTTTGATCA<br>TCCGATGGGCGGCTTGACAATCCCTTGTCCTGTAGATACTTTTATCAGTATAACATCTCAGCTCCAAGGATGAAGATGA<br>TGATGATCCAACAAAAATACATTAACAATTTTTTTTTTTTACTCAAACTAGAGATGGAGGAGTATTCCATGTGAATAGATA<br>ACATTTTTTTCTCCTCTTTCCTATTTGATAGAGTTTGTTCTAGACGATTAGAAAATTTCGAATGTGATTGCCGTTTTTGTAT<br>AACAAAGATCAAATCATTTACAAGTTGCGGTAGCTTTTTGCTTGATCCAATTGTTCAACGAGTATGAAACAAACTAATCA<br>AAGATATCACTTGACGACTTAGCAATCTAAATATCAGTTATTGCAAAAATGTAAAATGTCATAAAAGATCTCTTCGTTGC<br>AGCAAATCTTGAAACTGAGATTGGATTAGGAACGAAGATGGAACAAAGAATCTTTTATCTTCTCTATATTTATTTATATT<br>GAAAAACTCAAATGTAAAAATCAATACAAATATCAGATTACACAATCATGTCAGACTCTATATCATCAAGTTTGTTGGTC<br>GACGTCGATCAGTAAACAGAGACAGTTCAAGAGTAAAGAGAATCAAGTTTAAGCAATTTCACCACTACAGAAACATAA<br>TTATGTGACTATGATTAGCGTTATGATTAGGGTTAAGGTTAAGAGTATTATCCGGATCCGTGGCGCAATGGTAGCGCG<br>TCTGACTCCAGATCAGAAGGTTGCGTGTTCGATTCACGTCGGGTTCAAATCCCCCGAACAATAAATTCCGGAATATTTT<br>TTTACGTAGTGTCGGTCGGCACATTCAACATAGCGTTCGCCGTTAGGCTATTTCCAGCTTTGCCCTTTGCGTTTCAATTC<br>AACCTGTAATCTCTTAAGGGGCAGAGATAAGATTTCGACTGTCGATGATCTCTCCTTGAAAGAGTTCTGAAACAGTAAA<br>TTGTGATTTGCTTGAATTCCAAATTCAGTTATTTCTAGAGGACGAACAATCATGCAAGTATTTTTGCAACCATCCTTCTAT<br>GGATGCGGATAACGTAAACTCTAAGTCTCTAAGTCCATTCACGTTCATGGACACGGAGAGAACATCAGAGGTAAATG<br>GGGATGAATGTGCAGAGACAAATTAAGAAGGGCCATAAACGAATGGAAGTTTAATAAACGATGAGATTTATCCCCTTT<br>TTAAGACTTTCATTTAGATAAATCTTACCAAATTGATCCATGTTGGATTAGATTTCATTTTGGCGGGAACTTTTCCATTTTT<br>CAAATCAAACGAAAAAAGGAAGAAAAAAAAAAACTGAAAGTGAAATGAAACTAATATTCAACAGTAATTTGCGTGCTTAT<br>GAATATGATATATTTCAGTGTGTATTTGAAAATTTAAAATGGAAAATAACGAAAGGAAGTTACAGAAAAATCATAGTTAA
15	RB	Chr3:986547:991441	[illegible]<br>TTGGCGGAAGATCCATACACTATAAAAGTATAAAGAAAGAGCCCTACACTATATAGCAATAATTATCAGAATAGCTTTA<br>ACAAAATAATTATCAAGATATCGAAACATAATTACGTTGTTATCAACCTAGTCCGTTTAATAGATATACGATATTGTTTCC<br>TATTAAATTGGAAATTTCGGTTACAATCCGGATTCAATACACTTTTGACTTTTGATTTGGATTCGGTATAGTTTGGATAAA<br>CTAAAAAGTAAGGGGAATTATTTGTAATAAATAAAAGTAGAAGGATATTAGAAAAAAAATTCAAATTGTAACAAGTCGT<br>CTTTTTCTCTGTTCATAATAAAAAATTAAAAACCCTTGTCTTTCCAACTGTTGACCAAGACAAGCCCTCTCCACTTCAGAT<br>TGTTTCTAAAAGCAATTCTCAATCTTTTCTACACCGAAATAGCAACAAAATGAATTCAATCTCTTCATCTTCCATGCGTCG<br>TAATTGGATACACGATGTCTTCCCGAGCTTCCACGGTGCAGATGTCCGCAAATCGTTTCTGAGTCACACTCTGAAGGA<br>GCTCAGAAGCAAAGGTATCGACTTATTCATTGATAATGATATCTTGAGAGGAGAATTTATCGGCCCTGAGCTCAAAAAA<br>GCGATCCATGGATCGAGGCTCGCGATTGTCTTGCTCTCGAAAAGATACGCTTCTTCGTCGTGGTGTCTTGACGAATTG<br>GCGGAGATTATGAAGTGCAAGGAAGCTTTTGGTCAAACAGTGATGGCCATTTTCTACGAAGTGGATCCAACTGATGTA<br>GAGAAGCAGACCGGAGGTTTTGGGGAAGTCTTCACAGAAACTTGTGAGGGTAAAACAGCTGAGGACATTGAGAAAT<br>GGAGTCAAGCTCTTGCAAAAGTGGCAACTGTCACTAGTTACCTTTCAAGCAGCTGGTTCGTCCCTTTTAATTTCAACTTG<br>CTCTTGAACTTTCTGAACACATCAAACTTTGAAACGATATGCAGCCAAGAACTTGAGTTTGTGATTTTTGGATGACAATT<br>TAATCAGATGTATTCGTCTTTGTTAGGGATACTGAAACAGAAATGATCGAAGAAATTGCCACTGATGTTTCGAATATGTT<br>GACTAAGTCAACGCAATCAAGGGATTTCGTCGGCATAATTGGAATGGGATCTCATATGGAGAAGATGAAACCGTTGCT<br>ATGCCTAGAGTCAGAGGAAGTGAGGATGATAGGGATCTGGGGTCCTCCTGGAATTGGCAAGACCACCATTGCCAGA<br>TTTCTTTTCAACCAACTATTTGGCAATTTTGGTTTGATTGTCTTTATGGATAATATCAAAGCAATGTATACAATACCAATTG<br>GCTCAGATGACTATAGTGCAAAGTTGGATTTGCAGCAGAAGTTCATGTCTCAAATTATCAACGAAAAGGATATCAAGAT<br>TCCTCATTTAGGGGTTGCACAAGAAAGGCTCAAGGACGAGAAAGTTCTTGTTGTTCTTGATGGCGTTGATCAGTTATTT<br>CAATTGGAAGCCATGGCAAAAGAAACTCGGTGGTTCGGTCATGGAAGTCGTATTATCATTACAACACCAGATCTAAAA<br>CTTTTGAAGGCACATGGGATCACCCACATTTATAAGGTGGATTTTCCACCACCTGCTGAGGCTCTTGAAATCTTTTGTAT<br>GTATGCTTTCGAGCAAAAATCCCCTAAAGAGGGTTTCGAGATGCTTGCCTGGGAAGCTACAATTCTTGCTTGTCAACTC<br>CCTTTGGGACTAAGGGTTATAGGCTCTTATTTTCGAGGAAAGTACTCGAAGAAGATGTGGGAAGAGGCACTACCAGA<br>GTTAAGGAGTCACCTTGACGGAGAAATTGAAACCGTTTTAAAGTTCAACTACGATGCCTTATCGGAGAAAGATAAATC<br>GTTATTCCTTCATCTAACTTGCTTTTTCAACTTTGGAAGTATTGAGAATATAGAAGTTTATCTTGCCAAGACGTTCTCGGA<br>CTTCAGGCAACGGCTTGATCTCTTAGTTGAGAGATTTCTCATATCCATAGTCTCTGGATATTTAGAGATAAATCATTTGC<br>TAACCCTACTGGGAAGAGAAATTGTGCGAAAAAAATTATGGTAGTGAGGTCTCCATTCGTGAGCCTGGGAAACGCCA<br>GTTTTTGGTTGCTGCAGGAGATATTTGTCAAGTACTGAGTGATGATACAGCAGTAAGTTTTACTACTAATATTTCGTTGC<br>ATTGCTCCCTACAAGCTGAGTTTAATTGATAACTAACAGTTTATTTGATATATTGTATTGTATCCTTTTCAGGGTAGCAGC<br>ACTGTTATAGGAATAGATCTCACATTACCTGAGAAGGCCGACGAGGACGAAGAATTTTATATAAGTGAAAGAGCATTT<br>GAAAATATGACTAACCTCCAATTCTTAAAAATTAGTGGTGATTGCAGTAGATTGTACTTTCCGCCACGTCTGAACTCCAT
18	LB	Chr3:2642437:2646454	TTCGTCGCAGGCACGTCTTACAGTGGTTTACACCACAATATATCCTGCCAAGTACGAGTCAACTGCGAGAAGCTGGAC<br>ACAAAAGTACGCAATGGGTTGATGATATAAGACAAAGCAAAGGATATGTTATTGCGCATCGGAATAAAATAATAACTT<br>AAAAAAAATGCACTTGTTTGGTTTATCAAACGTTAGCAAGTATCGAACAACTTAGCTGCTCTTACTCCTCTTCTGTTCTGA<br>TCACTATAAACTAGTATTAATTTCGATTGTTGGGTCTACTTTTATTCTCTTGTTTCCCTCTCCTCACTTGGTTGCAGGAAAC<br>TGCAATTCTAATCGCCCCAAACGCAATGTTTGGACTGTTTTGGTACACTCTTGACATAAGGGGTTCCAATCTACACATG<br>GATCAACCACAACCAAAACCAAACCAACTATGAGCCTTCCATAGTTAGGATTATACATTGCTCCCAAATACAAAACTCG<br>TTCCTAATCTTCTATTAACTTATACCAAAGTTGGTATATTTATGATTAAATTATTGATTTTCTTAATTTTTTTAATATATTTCA<br>AAAGTGTACATGGTTCTAATAAAACACTTAAGAAATTTTCCATATTGTTTACAATAGCTGTTTTAGGAAATATCATATCTA<br>TAGACCAAACCAAGAATATCTTTAGACCAAACCAAGAATAGGTGTTTTAGAAAATAAACATATCTTTGGTTGCAATTTTC<br>CAATATCATCCGCCATGGGTCCCTACTAATAGGTCAGGCGGTACTTGCCGACAGAATCAGAAGCCTCGAGACTGACTT<br>GTATTCTCCGACTATAAAAAAGAAGTAGAGGGTTTTAGGCTTTTCACGAATCTCTTCTCTCTTTTGTAGTGGCCTTGAAA<br>AAAAGATTCAATCTCCTGTTGTGGTATGTTTCTTTTGATCCATCTTTCCTTTATGTTTTCTGTTTCAACTACTTCTTAGGGTT<br>TATCATCACTTACGTTACGTATGTATTGTTTTATTCGTCTTCCTCTATGATATGTTATGTTCTTAGTTGAATTCCTTAATACT<br>CATTGTGTGTTGCTTATCTCGTCCGCAGGTTTTCGAGTCTAAAAGCATGGCTTCTAAGAGGATTTCAAGGGAACTGAG<br>GGATATGCAGAGACATCCTCCAGCAAACTGTAGCGCAGGTAATCTCCCTTTTCTTGGCTGATTTTGACTGATAGATTCA<br>CACTAAAAACACTTTTAGGGTTTGATATATGTCACACTTTTATTTCATCAATCCACATTATATGTTTCTGAGTTTTTTTTGGT<br>AACGCTATGATTTGTTAGACTCTCTTTGGTTCTGTTATATATATATGTACTCTGCATGAACCTTTTTTTTTTTTTTTTTGTATTC<br>GAGAAAACTCGTTAAACCTTTGCCTGGTACGATTAAGAATATCCAGAGGTTTAGCTTGCTTTTATTTCAAGTGTCTCCTT<br>TTGTACTTCTTATTTATTCTGCCAATTTGTCTCTGTTCTATAAGAAGTATCACTTGATCATGCAGGTTTTATTGTCTTTTTCG<br>ATGAATAAATGGGTCTGATTGCAACGTCTTTCTTATAGGTCCTGTGGCTGAGGAGGACATATTCCATTGGCAAGCAACT<br>ATCATGGGACCGCATGACAGTCCGTATTCCGGAGGCGTATTTACGGTTTCTATTGATTTCTCTTCGGATTATCCTTTCAA<br>GCCACCAAAGGTGTTGTGTTTTTTCAACACTAATTTAATCATCTGATGTGTAATGAAAATCTATGATCATCAGGCTTATTA<br>GCGAGTGTACGATTTTGCAGGTGAATTTCAAGACTAAGGTGTACCACCCAAACATCGACAGCAAAGGAAGCATTTGCC<br>TTGACATATTGAAAGAGCAATGGAGTCCTGCTCCTACCACATCCAAGGTTTGAGTTTTGTCTCTCTACTACTATTTTTTAC<br>CAAAGTCATGTTCTGTAAAAAAAAAAACTAGTATATTGGTGAACTTTGTGCGAGTGATATTGATGGTGTGGAATTATATA<br>GGTTTTGTTGTCGATTTGCTCGCTGCTGACTGACCCGAACCCAAATGATCCTCTTGTGCCGGAGATAGCTCATCTCTAC

18	RB	Chr3:2636403:2642318	TGAAAGAGGACGTCAAAAACACAAACTGGTTACATAGCATCGATGACCGGTCGTCAGATCACGGACTCATGTTTGACA<br>ATGGAGGATTCGAGCTACTTCCAGAGCCATTTGCCCCACTATAACACACACACACACACACCCCACAAGTAAACACCTT<br>TTTTTCTTTCTAAAATCACTTCTCTTTTTTTTTTTTTTTTTTTTTAAATTTTATCTTACACAATTTGTTTGCTTGTCGATGGTGG<br>AAAGATATAAGGCTCAAGTGGTTCTTGGTTCTTTGGTTATTGATTCTTGATTCATGGCTATTGAGTGAGGTTTGTAACTT<br>TGTAGTTATAATATTTGAAGAGATGGTAACGTATAAGAGGGTACCTCATGTTCACTCTTGTTGGAAACGATCATGAACG<br>AAAAAACAATTAGAATGTGAAAAGAGGAAAGAGAGGGTCACACGACTCACGCGCCTAACGTGAATCAATGACAGAG<br>CTAAAGTTAATAATGGGTGACAACGAAGCACACAACCAACAACAGGCCAGTGTAGTTCTGATAAAAGAAAAAAATTAA<br>TTCCAGAAATTGACTTTCATTAATGTCATATTTGAGATTTAAGAAGTTTTAAATTTTTGTTATTTATTTTCTCTTTTATGTTAC<br>CCCAAAATAAATAAATACCCTCATTAATGTCACATACCAAACTCCTCCTCTTCAGGGGGTCCATATTTAAGAAAGATACC<br>CTCTTACCCGAATTAAAAATATCAAATAAAGTAAGCTCAATTTGCAACATATAATAATTTGTTGAATATTCCCCACTCTTG<br>TTGTTTTCCTTGCCCATTTTTTGATCTTGAAATACTTTTATCTTCTTATTGTTTAACGAATATCACAATCATCCATTTTCATTT<br>ATACATTGCATATTTTCAAAAAAGAAGCCATATATTTATTTAATCAACTGGTGACCCATTCAATAGAACATTACGATGCTT<br>TTAAAAGAATAAGGAAATTAAATGTTGGGAATTCAGTAAATGCCGTTTATTAAAAGCTCTTATGTATAAATTCATTTAGA<br>ATAAAAACAAAGAGAAATTAACAAAAGGACAAATATTTAATTGAGCTTAGCAATAAAAAAAAAATCTAAAAAATTCGGA<br>AGAGAGTCAAAGGGGGACTTAAAGCCAAAGAGAGCAAAGCAAATCGGAGTACAGAGAAGAAGAAGAGAGATTGGA<br>ATCTGAAACTGTAACATCGAGATTTACGTTTCTCCGTCGGCGTCACGCCGAAGCTTCCATACTCCAGAAACCCCAAAAC<br>GCTTATTTCTCAAAAATCTCCACTCTCTCTCTTCTGAATCTTCAATCACTTGGGGTTGTGTCACTGAATCTGCATCAATGG<br>CGATTCCAGATTCCTCACTCTTTTCTGCTTTTGACTTTAGCTCTAGCTTCTTCTTCACGAAATGCCGTTGTTGTTAAAAAGT<br>GATTTCTCCACTATCCCATTTCCTTTGGCATTTATTCTCCACTCTCTCTCACTTGTCTTCTTCTTCTTCTACGATCAGCTCAC<br>CAATGTCCTGAACCATTCTCCTCTGAATCAACCTCTAAGGTCTCTCTCACTTTCGATCTCTGTTTTCCCTCTTGTTTTGGG<br>GTTTATTAGCTTCGTTGTTTATTCCTTTAAAAGATTTCTTCTTTCTCAATTTCAATTACTCCGTTTAGCTTAGCTGGAGAAA<br>CCCTAGTTTGTAGATTGCAGATCTGGGTTAATCAAGTATCTGTGCACACATTTATGTCTAAAATTAGTTACTTTCGTAATT<br>GGATTTGTTTTTTTTGTGAAGATTTATTATTTGAAGTCAAACAGATTTCAGCTGATTGAATCAATGTGTGCATATAGCTTC<br>CATAATTTCATATTTTGATTTGAGAATCGAGTAATGTTGCTTTGGACTTTATCATCAGCATTCCCACTTTGGTTTGAGAAA<br>TTACGCTATTGTATCTTTGTCATTATTAAGGAAGCTTTTCGTTGTGTTGATGATCTTTTTACTCATGATACCTGTGATGTGT<br>AGGTGTCATTCTTGGCTACTTTATCCATAGATACATTTCTCATATTGAGTTTAGCTTTTATGTGGCTATAAATCATTACATG<br>ATGTGTTTTGTGTTCTCTTGCAGTTGCTGTTGAATGAGAAAGCTTTGATGTGAGCGAGCCTGAGAGTGTAACAAAGGA<br>CTGATGATGAAGATTATTGCGGCGTTTCTCTTCCTCTTGGTCACGACTTTTGTTTCCCGTTGTTTGTCTGCAGACATAGA<br>ATCGGACAAGCAAGCACTGCTTGAGTTTGCATCTCTTGTTCCTCACAGTCGCAAACTCAACTGGAACTCCACTATTCCA<br>ATCTGTGCCTCTTGGACTGGTATCACCTGCTCCAAGAACAACGCCCGTGTAACCGCACTCCGTTTGCCTGGATCTGGA<br>CTTTATGGACCTTTACCAGAGAAAACATTTGAGAAGCTTGATGCCCTCAGGATCATTAGCCTTAGATCCAACCACCTTC<br>AAGGGAACATTCCATCTGTCATTCTTTCACTTCCTTTTATCCGATCACTATACTTTCATGAAAACAACTTTTCTGGTACTAT
19	LB	Backbone	AAGGAGTCGTGAGTTCTGAGCATTGGTCGGTAACAAGATGCTATGCATTTCTTGTCTCCCCTATCCCCCCGTTACTTG<br>GACTGGTTCCCATGCGTTACCGTAGACCGATCCACCTTACACTTCGCTTTACAATAGCCTCGTGAATAATCAGGGTGAC<br>TCAGACACAGTAGGAGACTTACGCCAGACGTGACAGGATATATTGGCGGGTAAACTAAGTCGCTGTATGTGTTTGTTT<br>GCAGCCGTGCGGCTGCATGAAATCCTGGCCGGTTTGTCTGATGCCAAGCTGGCGGCCTGGCCGGCCAGCTTGGCCG<br>CTGAAGAAACCGAGCGCCGCCGTCTAAAAAGGTGATGTGTATTTGAGTAAAACAGCTTGCGTCATGCGGTCGCTGCG<br>TATATGATGCGATGAGTAAATAAACAAATACGCAAGGGGAACGCATGAAGGTTATCGCTGTACTTAACCAGAAAGGC<br>GGGTCAGGCAAGACGACCATCGCAACCCATCTAGCCCGCGCCCTGCAACTCGCCGGGGCCGATGTTCTGTTAGTCG<br>ATTCCGATCCCCAGGGCAGTGCCCGCGATTGGGCGGCCGTGCGGGAAGATCAACCGCTAACCGTTGTCGGCATCGA<br>CCGCCCGACGATTGACCGCGACGTGAAGGCCATCGGCCGGCGCGACTTCGTAGTGATCGACGGAGCGCCCCAGGC<br>GGCGGACTTGGCTGTGTCCGCGATCAAGGCAGCCGACTTCGTGCTGATTCCGGTGCAGCCAAGCCCTTACGACATAT<br>GGGCCACCGCCGACCTGGTGGAGCTGGTTAAGCAGCGCATTGAGGTCACGGATGGAAGGCTACAAGCGGCCTTTGT<br>CGTGTCGCGGGCGATCAAAGGCACGCGCATCGGCGGTGAGGTTGCCGAGGCGCTGGCCGGGTACGAGCTGCCCAT<br>TCTTGAGTCCCGTATCACGCAGCGCGTGAGCTACCCAGGCACTGCCGCCGCCGGCACAACCGTTCTTGAATCAGAAC<br>CCGAGGGCGACGCTGCCCGCGAGGTCCAGGCGCTGGCCGCTGAAATTAAATCAAAACTCATTTGAGTTAATGAGGTA<br>AAGAGAAAATGAGCAAAAGCACAAACACGCTAAGTGCCGGCCGTCCGAGCGCACGCAGCAGCAAGGCTGCAACGTT<br>GGCCAGCCTGGCAGACACGCCAGCCATGAAGCGGGTCAACTTTCAGTTGCCGGCGGAGGATCACACCAAGCTGAAG<br>ATGTACGCGGTACGCCAAGGCAAGACCATTACCGAGCTGCTATCTGAATACATCGCGCAGCTACCAGAGTAAATGAG<br>CAAATGAATAAATGAGTAGATGAATTTTAGCGGCTAAAGGAGGCGGCATGGAAAATCAAGAACAACCAGGCACCGAC<br>GCCGTGGAATGCCCCATGTGTGGAGGAACGGGCGGTTGGCCAGGCGTAAGCGGCTGGGTTGTCTGCCGGCCCTGC<br>AATGGCACTGGAACCCCCAAGCCCGAGGAATCGGCGTGAGCGGTCGCAAACCATCCGGCCCGGTACAAATCGGCG<br>CGGCGCTGGGTGATGACCTGGTGGAGAAGTTGAAGGCCGCGCAGGCCGCCCAGCGGCAACGCATCGAGGCAGAA<br>GCACGCCCCGGTGAATCGTGGCAACCGGCCGCTGATCGAATCCGCAAAGAATCCCGGCAACCGCCGGCAGCCGGT<br>GCGTCGTCGATTAGGAAGCCGCCCAAGGGCGACGAGCAACCAGATTTTTTCGTTCCGATGCTCTATGACGTGGGCAC<br>CCGCGATAGTCGCAGCATCATGGACGTGGCCGTTTTCCGTCTGTCGAAGCGTGACCGACGAGCTGGCGAGGTGATC<br>CGCTACGAGCTTCCAGACGGGCACGTAGAGGTTTCCGCAGGGCCGGCCGGCATGGCCAGTGTGTGGGATTACGAC<br>CTGGTACTGATGGCGGTTTCCCATCTAACCGAATCCATGAACCGATACCGGGAAGGGAAGGGAGACAAGCCCGGCC<br>GCGTGTTCCGTCCACACGTTGCGGACGTACTCAAGTTCTGCCGGCGAGCCGATGGCGGAAAGCAGAAAGACGACCT<br>GGTAGAAACCTGCATTCGGTTAAACACCACGCACGTTGCCATGCAGCGTACGAAGAAGGCCAAGAACGGCCGCCTG<br>GTGACGGTATCCGAGGGTGAAGCCTTGATTAGCCGCTACAAGATCGTAAAGAGCGAAACCGGGCGGCCGGAGTACA<br>TCGAGATCGAGCTAGCTGATTGGATGTACCGCGAGATCACAGAAGGCAAGAACCCGGACGTGCTGACGGTTCACCC<br>CGATTACTTTTTGATCGATCCCGGCATCGGCCGTTTTCTCTACCGCCTGGCACGCCGCGCCGCAGGCAAGGCAGAAG
19	RB	Chr1:4946719:4948952	AGCCACATTCTCCACTATCTATACGCTACATACCAGAGTTACAAAATCATTTGAATAGAGGATTAAACTTGGGGTGAGA<br>AGCATGTAATTTGATCAAGACCATTGATTCCTTCAGGCATTTTAAAAGCTATATAACCAACTGCACCCAAATGATGCAAA<br>GGAGCTCACCAAAAATACGATCTTGCACAACATTCACTACATTCTCAATCTATCCTCTCTTTCAAGTGTTCTTTTGATCAC<br>AATATACAAAAGCTAAGCAAGACAATACTTTGATCAAGATTCAAGACAAATTCACAAACTTAAGCTGGAGCTAACTACG<br>CCAAATCTTAGAGATAACAAATGGAAAGAAACTAAGGATCACTTTGCAATAAAAACATTTCACAACATTCTCAGGTTTAA<br>GCGAGTTATACAAAAACAAAAGAAATCAGTTTTATATGTAAGCATAAAAACTAAGCACTCATACACTAACTAAACGCAA<br>AGATCAATTTAACATAGACGAGAAACTACATCTCTACGTTCCAATCAAACGAAGAAAAATTCAAAATTAGCGACACAAA<br>ATGAATCAATCATCAAAGATTTACCTCATCGAAGAAGAAAGATGCTCTTCAGTGAGACGGAGCAGTCTGTTCCTGGTG<br>GTGGTGAGATGATTTGGAAGGAGCCAAAGCTTTGTTGTAGATCTGCTCACCGACGAGATAAACGCAGAAGGCGGCG<br>ACGCCGATTCCAAGTCCGGGAAGAGCGTGTCTCATTTGATTCGAAAGCATCGGATGCTTCCTCCATTCATCTCTTCGCC<br>GGAAAAATTCGCCGGTTGTTCCCAAAGGCTTCGCCATTTTCTCAGGTATTCGATTAATCTCCTGCGACCGTACTATTCCT<br>CTTAACTTTCTTCTCAAATGACAAATCATGGGCTTCTTTGATGTATATGGGCTTTAAATTTATTTTTTGTCGTCATATGGGC<br>TTTAACTTTAGGTTATCATTTGGGCTTCTTTTGAAAAAAAATCGGAAAATAATAATGTCGTAAACTAGTTTTCTTATGGTT<br>CTCTTTAATATCAAAATATAATGTCGGAAATAAGTAATTAAGTTTAATAATATTGCATAGCATCATATATAAATTATATCAG<br>AAATCTATCATTGTAGAGTTGTTATTATTTATTTCTTGTTAAACAGAAAATAAATACAAATGGTAAATCATAGTGAAACAT
21	LB	Chr3:19877281:19877456	CTGAGTAGTTTGTTCTTATTGCAGCGTACTACCGTGGAGCCATGGGGATTTTGCTTGTGTATGATGTGACTGATGAATC<br>ATCTTTCAACAGTAAGTACTCTCTTTACTTTTTACTTGCAGTCCTTATGCCACCCAACAAGCTCTTGTAAACCACTGTAAG
21	RB	Chr3:19876141:19877280	ATTTGTTTGAATAAAAGGTTTCAATTCTTGCAATGAGTGATAAAACATTAATAGTTGAAAGTTTGAAACAGAACCATTCT<br>CGAATGTATACATAGAATCCAATATCATTGGTGTGTGAGTGAGATAAACTGATATTATCATAAGTCATAATCTGATGTCA<br>GAGAATCCATTCCAGAAATATGGAATACGAATTGTGTCCTTCACAGTCACACTATACCATTAGGGAAAGAGAGAATCTT<br>AAAAGAATACGTAGAAACACCATCTCATATAGAAGAAAAGCAAAAGAAGATTGATTTCCTTTTTCATTTTTTCTTTCCAAA<br>TACCATTTCTCTTTCACTAGCCAGATTCTCTCCTTTGACAGGCGTCGTGGACCCAATTTTTATGTGCCGCAACATGCTGA<br>TTTCTGAGTAGCTTGAGATGTTCCCGCACCTTGGTCGGATTGGTTGATTTTGATTGTTTGCGGCTACAAAAATGATAAG<br>AAATTCGATGACGTTAGTAAGGTTGATACACATACTGAAATGTGATTTTTACCACAATTGCAATGTATGAATGATGACA<br>GTGGAAATTAATTCTATAGCTCAAACGAACCTCAGCTCGTGCATCGGTATCTGCAAGTCTTTGCTTAATGTCTTTAGCAA<br>TAGAGAAGAAAACTTCCTCAACGTTTAAGTTAGTCTTGGCACTCTGCAATTAGCAAACTCATCAAAGTTAACCATTTAAA

Map to same locus
Map to different locus/ backbone

Supplementary Table 1: Plasmids used in this study

Plasmid	Figure	Description(s)
**pWS9500**	Fig. 1c,d; Fig 1e-i; Fig. 2a-c; Extended Data Fig. 7d,e,g	Divergent; Wild type; Original; tHSP
**pWS9501**	Fig. 1c,d, Extended Data Fig. 7d	Reverse
**pWS9504**	Fig. 1c,d, Extended Data Fig. 7d	Forward
**pWS9505**	Fig. 1c,d, Extended Data Fig. 7d	Convergent
**pWS9507**	Fig. 2a-c	Border Var 1
**pWS9508**	Fig. 2a-c	Border Var 2
**pWS9509**	Fig. 2a-c	Border Var 3
**pWS9510**	Fig. 2a-c	Border Var 4
**pWS9993**	Fig. 2a-c	Border Var 5
**pWS9512**	Fig. 2a-c	Border Var 6
**pWS9966**	Fig. 2d-i	pRB-Max
**pWS9967**	Fig. 2d-i; Fig. 3e,f; Fig. 5a	pRB-Mid; pRB-Mid (WT); pRB-Mid (1xLB)
**pWS9968**	Fig. 2d-i	pRB-Min
**pWS9925**	Fig. 3c,d	pRB-Mid (WT)
**pWS9928**	Fig. 3c,d	pRB-Mid (R106H)
**pWS8872**	Fig. 3c,d	pCAMBIA (WT)
**pWS8871**	Fig. 3c,d	pCAMBIA (R106H)
**pWS10045**	Fig. 3e,f	pRB-Mid (R106H)
**pWS8883**	Fig. 3e,f	pCAMBIA (WT)
**pWS8882**	Fig. 3e,f	pCAMBIA (R106H)
**pWS9474**	Fig. 4a,b	35S
**pWS9475**	Fig. 4a	pACT2
**pWS9476**	Fig. 4a	pACT8
**pWS9478**	Fig. 4a	pAGP15
**pWS9479**	Fig. 4a	pEF1A2
**pWS9480**	Fig. 4a	pEF1A3
**pWS9481**	Fig. 4a	pEF1A4
**pWS9482**	Fig. 4a	pLEA26
**pWS9483**	Fig. 4a	pPLDA1
**pWS9484**	Fig. 4a	pTUB6
**pWS9485**	Fig. 4a	pUBQ1
**pWS9486**	Fig. 4a	pUBQ4
**pWS9487**	Fig. 4a	pUBQ11
**pWS9605**	Fig. 4a,b	nos
**pWS9696**	Fig. 4a,b	Negative control
**pWS9682**	Fig. 4b	t35S
**pWS9683**	Fig. 4b	tAGP15
**pWS9684**	Fig. 4b	tAPX1
**pWS9685**	Fig. 4b	tEF1A1
**pWS9686**	Fig. 4b	tEF1A2
**pWS9687**	Fig. 4b	tFAD2
**pWS9688**	Fig. 4b	tGADPH
**pWS9689**	Fig. 4b	tUBQ4
**pWS9690**	Fig. 4b	tUBQ10
**pWS9691**	Fig. 4b	tUBQ11
**pWS10032**	Fig. 4c	p35S
**pWS10033**	Fig. 4c	pEF1A3
**pWS10068**	Fig. 4c	pUBQ10:RUBY positive control
**pWS10034**	Fig. 4c	pEC:RUBY negative control
**pWS9994**	Fig. 4d	p35S:bar
**pWS9995**	Fig. 4d	pEF1A3:bar
**pWS9515**	Fig. 5a	pRB-Mid, 1xLB
**pWS9519**	Fig. 5a	pRB-Mid, 2xLB
**pWS10146**	Fig. 5b	pRB-Mid-2LB
**pWS9498**	Extended Data Fig. 7f	Divergent
**pWS9499**	Extended Data Fig. 7f	Reverse
**pWS9502**	Extended Data Fig. 7f	Forward
**pWS9503**	Extended Data Fig. 7f	Convergent
**pWS9473**	Extended Data Fig. 7e	Recoded
**pWS9464**	Extended Data Fig. 7e	Linker
**pWS9838**	Extended Data Fig. 7e	Intron
**pWS10143**	Extended Data Fig. 7g	tFAD2
**pWS10144**	Extended Data Fig. 7g	Tnos
**pWS10145**	Extended Data Fig. 7g	Tocs
**pWS10069**	Extended Data Fig. 13	p35S
**pWS10070**	Extended Data Fig. 13	pEF1A3
**pWS10071**	Extended Data Fig. 13	Pnos
**pWS10072**	Extended Data Fig. 13	No promoter
**pWS9997**	Extended Data Fig. 14	pEC:StayGold-NLS:tHSP
**pWS10150**	Extended Data Fig. 15	pEF1A3:nptII:tUBQ11

Supplementary Table 2: T-DNA plasmid toolkit

Plasmid	Description
**pWS10205**	Plant Entry Vector
**pWS10206**	Expansion module
**pWS10207**	1F assembly cassette
**pWS10208**	2F-T assembly cassette
**pWS10209**	2F assembly cassette
**pWS10210**	3F-T assembly cassette
**pWS10211**	3F assembly cassette
**pWS10212**	4F-T assembly cassette
**pWS10213**	4F assembly cassette
**pWS10214**	5F-T assembly cassette
**pWS10215**	5F assembly cassette
**pWS10216**	6F-T assembly cassette
**pWS10217**	6F assembly cassette
**pWS10218**	7F-T assembly cassette
**pWS10219**	1R assembly cassette
**pWS10220**	2R-T assembly cassette
**pWS10221**	2R assembly cassette
**pWS10222**	3R-T assembly cassette
**pWS10223**	3R assembly cassette
**pWS10224**	4R-T assembly cassette
**pWS10225**	4R assembly cassette
**pWS10226**	5R-T assembly cassette
**pWS10227**	5R assembly cassette
**pWS10228**	6R-T assembly cassette
**pWS10229**	6R assembly cassette
**pWS10230**	7R-T assembly cassette
**pWS10231**	1 assembly spacer
**pWS10232**	2-T assembly spacer
**pWS10233**	2 assembly spacer
**pWS10234**	3-T assembly spacer
**pWS10235**	3 assembly spacer
**pWS10236**	4-T assembly spacer
**pWS10237**	4 assembly spacer
**pWS10238**	5-T assembly spacer
**pWS10239**	5 assembly spacer
**pWS10240**	6-T assembly spacer
**pWS10241**	6 assembly spacer
**pWS10242**	7-T assembly spacer
**pWS10330**	pRB-Mid-2LB
**pWS10331**	pRB-Mid-2LB bar
**pWS10332**	pRB-Mid-2LB nptII
**pWS10333**	pRB-Mid-2LB hpt

Supplementary Table 3: Arabidopsis promoter design

Name	Gene	Intergenic	Orientation	Other notes	Acceptable	Promoter + 5'UTR	Final notes
ACT2	AT3G18780	Yes			Yes	1300	Tested
ACT8	AT1G49240	No	Tandem		Yes	1040	Tested
AGP15	AT5G11740	Yes			Yes	658	Tested
APX1	AT1G07890	No	Divergent		No		
ARF1	AT1G23490	No	Tandem		Yes	759	Did not test, synthesis failure
AVP1	AT1G15690	No	Divergent		No		
EF1A1	AT5G60390	No	Divergent		No		
EF1A2	AT1G07940	No	Tandem		Yes	1000	Tested
EF1A3	AT1G07920	No	Tandem		Yes	1000	Tested
EF1A4	AT1G07930	No	Tandem		Yes	966	Tested
GADPH	AT1G13440	No	Divergent		No		
HTR5	AT4G40040	No	Divergent		No		
LEA26	AT2G44060	No	Tandem		Yes	827	Tested
PCC1	AT3G13920	No	Divergent		No		
PLDA1	AT3G15730	Yes			Yes	1163	Tested
SHM4	AT4G13930	No	Divergent		No		
TPI	AT3G55440	No	Divergent		No		
TUB2	AT5G62690	No	Divergent		No		
TUB6	AT5G12250	Yes			Yes	1100	Tested
UBC10	AT5G53300	No	Divergent		No		
UBC9	AT4G27960	No	Tandem	Alternative splicing in 5' UTR	No		
UBQ1	AT3G52590	No	Tandem		Yes	683	Tested
UBQ10	AT4G05320	No	Divergent		No		
UBQ11	AT4G05050	No	Tandem		Yes	720	Tested
UBQ4	AT5G20620	No	Tandem		Yes	790	Tested

Selected for charatcerisation

Promoter selection criteria
1. Not in divergent orientation with neighbouring gene, including lncRNA (within 2 kb)
2. No 5' UTR overlap with neigbouring gene body
3. No alternative splicing in 5' UTR

Promoter region defined by open chromatin in leaf ATAC-seq data from Lu, Z. et al. The prevalence, evolution and chromatin signatures of plant regulatoryelements. Nat Plants 5, 1250–1259 (2019).

Supplementary Table 4: Arabidopsis terminator design

Name	Gene	3' UTR (TAIR10)	Termination Window (Mo et al. 2021)	Longest Read-through (Mo et al. 2021)	Intergenic	Orientation	Notes	Acceptable	Selection	Final notes
ACT2	AT3G18780		165	377	No	Tandem	Promoter for next gene	No		
ACT8	AT1G49240		123	281	No	Tandem	Promoter for next gene	No		
AGP15	AT5G11740	460	196	**492**	No	Convergent		Yes	542	Tested
APX1	AT1G07890	230	181	**689**	No	Convergent		Yes	694	Tested, accidentally added 5 bp instead of 50 bp
ARF1	AT1G23490		226	645	No	Tandem	Promoter for next gene	No		
AVP1	AT1G15690		155	389	No	Convergent	Overlap with next gene	No		
EF1A1	AT5G60390	**345**	153	308	No	Convergent		Yes	395	Tested
EF1A2	AT1G07940	**401**	124	245	Yes			Yes	451	Tested
EF1A3	AT1G07920		134	223	No	Tandem	Promoter for next gene	No		
EF1A4	AT1G07930		117	251	No	Tandem	Promoter for next gene	No		
FAD2	AT3G12120	**341**	120	313	Yes	Convergent		Yes	391	Tested
GADPH	AT1G13440	**388**	144	**388**	No	Convergent		Yes	438	Tested
HTR5	AT4G40040		102	263	No	Tandem	Promoter for next gene	No		
LEA26	AT2G44060		153	295	No	Tandem	Promoter for next gene	No		
PCC1	AT3G13920		131	351	No	Tandem	Promoter for next gene	No		
PLDA1	AT3G15730		225	577	No	Convergent	Overlap with next gene	No		
SHM4	AT4G13930		357	1092	No	Covergent	Too long	No		
TPI	AT3G55440		221	510	No	Convergent	Overlap with next gene	No		
TUB	AT5G62690		129	254	No	Tandem	Promoter for next gene	No		
TUB2	AT5G62690		129	254	No	Tandem	Promoter for next gene	No		
TUB6	AT5G12250		142	239	No	Tandem	Promoter for next gene	No		
UBC10	AT5G53300		126	315	Yes		Intron in 3' UTR	No		
UBQ1	AT3G52590		162	388	No	Convergent	Overlap with next gene	No		
UBQ10	AT4G05320	376	128	**416**	No	Convergent		Yes	466	Tested
UBQ11	AT4G05050	267	170	**507**	Yes			Yes	557	Tested
UBQ4	AT5G20620	262	156	**304**	No	Convergent		Yes	354	Tested

Selected for charatcerisation

Terminator selection criteria
1. Not in tandem orientation with downstream neighbouring gene, including lncRNA (within 2 kb)
2. No 3'UTR overlap with neigbouring gene body
3. Does not contain intron

3' UTR from TAIR10 annotation
Termination window and longest read-through from Mo, W. et al. Landscape of transcription termination in Arabidopsis revealed bysingle-molecule nascent RNA sequencing. Genome Biol 22, 322 (2021).

Supplementary Table 5: Oligonucleotides used in this study

Description	Forward primer (5' to 3')	Reverse primer (5' to 3')
RUBY cDNA for RT-qPCR expression analsysis	CTACCGTGATCAAGAATGGATC	CAGCTTGGTGGTCTGCTG
UBC9 cDNA for RT-qPCR expression analsysis	TCACAATTTCCAAGGTGCTG	GTGGACTCGTACTTGTTCTTGTC
Arabidopdis thaliana genome for qPCR T-DNA copy number analysis	GGAACATCCTATTCTACTTACCGAG	AACAGCCTGAATAGCCACATAC
RUBY CDS for qPCR T-DNA copy number analysis	CACTCATTTCCCTCAGACTCG	CACTATTCGGGATCGTGC
Agrobacterium genome for qPCR T-DNA plasmid copy number analsysis	GAGTACCGGAATCTCGTCAAAGCC	CGAAGATCTCTACGGCAACTACCTGG
T-DNA plasmid backbone for qPCR T-DNA plasmid copy number analsysis	GATCATCCTGATCGACAAGACCGG	CTGCCGAGAAAGTATCCATCATGGC
Low-cycle PCR detection of T-DNA - bar	GAACAGGCAAAGAGAAATCG	GCTGATATCCGTAGAGCTACTG
Low-cycle PCR detection of T-DNA - RUBY	GTTCGGCTTCGACATCAAC	TCAAGTTTGGCCTGCATG
Low-cycle PCR detection of T-DNA - Backbone	CGTGAGTTTTCGTTCCACTG	CTTACCGGATACCTGTCCG
Low-cycle PCR detection of T-DNA - LB read-through	CCACCACTTCAAGAACTCTGTAG	TGCTCAGAACTCACGACTCC
iPCR genomic DNA amplification - 1.7 kb	AACCGCGTTATTTTATATGTGCC	GCCGATGTTTTCCAATCCC
iPCR genomic DNA amplification - 3.0 kb	GCATGGACCGTTCTCTATTACAG	TCGAATAGATCGTGTAATGGTTATTTGG
iPCR genomic DNA amplification - 4.8 kb	CATCTTTATGTCCTTAAAATCGTTGGG	AGGAATACTCTCCCTTGGTCA
Inverse PCR mapping at LB (Temporary primers)	TTCAGCACCACGAAATAGTAGC	CAGTCCCGAGCCGTCTTACTC
Inverse PCR mapping at RB (Temporary primers)	CCTCGTGAATAATCAGGGTGAC	CTATTGTAAAGCGAAGTGTAAGGTG
Inverse PCR mapping at LB (Final barcode primers)	TTCGGGAGCGGATTATACAC	CGGATCGAACTTAGGTAGCC
Inverse PCR mapping at RB (Final barcode primers)	ACAAGGAGTCGGCATATCAC	AGACAAGCCTTAACCGTAGG
